# Local recurrence rates of superficial versus deep soft tissue sarcoma

**DOI:** 10.1007/s00402-024-05326-1

**Published:** 2024-06-08

**Authors:** James S. Lin, Lisa Coleman, Ryan T. Voskuil, Azeem Malik, Joel L. Mayerson, Thomas J. Scharschmidt

**Affiliations:** 1 Department of Orthopaedics, The Ohio State University Wexner Medical Center, Columbus, Ohio United States; 2 Department of Orthopaedic Surgery, Division of Musculoskeletal Oncology, The James Cancer Hospital and Solove Research Institute, Division of Musculoskeletal Oncology, The Ohio State University Wexner Medical Center, Nationwide Children’s Hospital, Columbus, Ohio United States

**Keywords:** Sarcoma, Soft tissue, Recurrence, Superficial lesions, Resection margin

## Abstract

**Introduction:**

Soft tissue sarcomas are a group of malignancies that commonly occur in the extremities. As deep lesions may exist within the confines of the muscular fascia, we postulate that local recurrence rates are higher for superficial soft tissue sarcomas managed by the standard of care.

**Materials and methods:**

A retrospective review was performed on 90 patients who underwent surgical resection of soft tissue sarcomas of the extremity from 2007 to 2015. Patients with minimum 2-year follow-up and adequate operative, pathologic, and clinical outcomes data were included.

**Results:**

Mean age was 54 ± 18 years with 49 (54.4%) patients being male. Lesions in 77.8% of cases were deep, and 22.2% were superficial to fascia. Following the index surgical resection, a total of 33 (36.7%) patients had positive margins. A total of 17 (18.9%) patients had a local recurrence. Overall, 3-year survival was 92.7%, and 5-year survival was 79.0%. Five-year recurrence-free survival of deep sarcomas was 91.1% versus 58.2% of superficial lesions (*p* = 0.006). Patients with higher tumor depth had lower odds of experiencing a local recurrence (HR 0.26 [95% CI 0.09–0.72]). Local recurence rates was also associated with positive surgical margins on initial resection (33.3% versus 12.3%) (*p* = 0.027).

**Conclusions:**

In this series, superficial tumor depth was associated with local recurrence of soft tissue sarcomas of the extremity following surgical resection. Positive surgical margins was also associated with local recurrence.

**Supplementary Information:**

The online version contains supplementary material available at 10.1007/s00402-024-05326-1.

## Introduction

Soft tissue sarcomas are a heterogeneous group of rare malignancies that often occur in the extremities [14, 20]. These lesions usually present as enlarging, painless masses, and initial work-up should consist of radiographs and contrast-enhanced magnetic resonance imaging (MRI) as the imaging of choice to characterize these tumors in terms of localization, staging, and their relationship to neurovascular structures (9,20,29). If MRI is indeterminate or concerning for sarcoma, a biopsy is obtained to confirm the diagnosis – ideally performed by or under the direction of the treating oncologic surgeon [20]. Typically, lesions are surgically managed with wide resection, with limb salvage being possible in over 90% of patients [25].

Radiation is also commonly used as an adjunctive treatment. A randomized prospective trial by Yang et al. [35] found that postoperative radiation after limb-sparing surgery of soft tissue sarcoma of the extremity was associated with decreased local recurrence compared to surgery alone, although there was no difference in overall survival. These authors maintain that adjuvant radiation therapy should be selective, as patients with a low risk of local recurrence following surgery may not require it [3, 35]. This is supported by other authors who have contended that surgery alone without local adjuvant therapy is adequate for superficial soft tissue sarcoma regardless of tumor grade, with local recurrence rates of less than 10% [27], as radiation therapy is associated with wound complications and toxic effects on the skin [22].

The extent that local recurrence influences overall survival is also mixed in the literature [30, 35]. Some studies have reported that local recurrence is not adversely associated with survival [24, 31], while others have found that local recurrence to be an independent predictor of decreased survival [10, 11, 21]. Nevertheless, local recurrence can potentially lead to considerable morbidity and should be avoided if possible [21]. Existing literature has consistently reported that positive surgical margins is a key contributing factor to local recurrence, along with tumor size and grade [4, 13, 17, 28, 31, 36].

Depth of tumor may also play a role in clinical outcomes and management. Soft tissue sarcomas classified by depth is based on the tumor’s location in relation to the deep muscle fascia. Specifically, superficial soft tissue sarcomas lie above the deep fascial layer and can include the skin, whereas deep soft tissue sarcomas lie deep to or involve the fascial layer [2, 4, 6, 26, 27]. Some authors have contended that superficial soft tissue sarcomas have better prognosis compared to deep lesions [6, 12, 19, 32], although findings have been mixed in the literature [23]. Authors have suggested that the superficial nature of these lesions may afford earlier detection and management, which in turn may lead to improved survival [32]. Some studies have also reported that superficial soft tissue sarcomas are less likely to have local recurrence than their deep counterparts, although there may be little difference when examining cumulative probabilities of local recurrence up to ten-years as death becomes a competing end point [4]. Existing data of superficial soft tissue sarcomas of the extremities suggest local recurrence rates around 11% and 19.7% [2, 5, 18].

We undertook this study to investigate the rates of local recurrence for soft tissue sarcomas of the extremity treated at our institution. Since superficial soft tissue sarcomas are not bound by muscular fascia as deep lesions may be, we postulate that local recurrence rates are higher for superficial soft tissue sarcoma than previously reported. In addition, we hypothesize that recurrence rates of superficial lesions are higher than that of their deep counterparts managed by the standard of care.

## Methods

Following institutional review board (IRB) approval, we performed a retrospective review of patients who had undergone surgical resection of soft tissue sarcoma of the upper or lower extremity from 2007 to 2015 at a single university hospital by two fellowship-trained musculoskeletal oncology surgeons. Candidate patients were identified by current procedural terminology (CPT) codes for resection of lesions in the extremity. Charts were then manually reviewed for diagnosis of soft tissue sarcomas, and patients with minimum two year follow-up and adequate operative, pathologic, and clinical outcomes data were included for analysis.

Data collected included demographic characteristics of patients, comorbidities, sarcoma subtype, location of lesion, depth of invasion, tumor grade, tumor size, surgical margins, presence of metastatic disease at presentation, use of adjunctive treatment modalities at index presentation (chemotherapy and/or radiation), disease free survival (i.e. no local recurrences), and overall survival. Final determination of tumor depth was made from intraoperative findings. Presence of metastases were assessed for with staging studies, commonly with computed tomography (CT) scans of the chest. Whole body bone scans were obtained when there were concerns for osseous metastases, and specific imaging was performed for pathologies with predilections for metastases to certain areas (e.g. brain MRI obtained for alveolar soft part sarcoma). Positive margins were considered R1 or R2 as defined by R-classification of residual tumor on resection margin [33]. Continuous variable data were reported as means and standard deviations (±) from the mean. Categorical variable data were reported as frequencies with percentages. Kaplan-Meier log-rank tests were used to assess for significant differences in unadjusted 5-year recurrence free survival, based on tumor depth. A Cox-regression hazard model, that utilized a backward step-wise approach with exclusion at *p* = 0.1, was employed to assess whether tumor depth was associated with differences in local recurrence, after controlling for all baseline demographics. All statistical analysis was performed using SPSSv24 (Armonk, NY, USA). For all statistical purposes, a p-value of less than 0.05 was considered significant.

## Results

A total of 90 patients, 49 (54.4%) men and 41 (45.6%) women, undergoing resection of soft tissue sarcoma of the extremity met our inclusion criteria (Table 1). The mean age for the sample was 54 ± 18 years (range 16 to 92). Mean post-operative follow-up was 5.2 ± 2.1 (range 2.0 to 10.3) years. The most common type of sarcoma was liposarcoma in 20 (22.2%) cases, followed by myxofibrosarcoma in 16 (17.8%) cases and undifferentiated pleomorphic sarcoma/malignant fibrous histiocytoma in 11 (12.2%) cases. Sixty-three (70.0%) tumors were located in the lower extremity, followed by 17 (18.9%) in the upper extremity and 10 (11.1%) in the hip/pelvis. The tumor was deep to the fascia in 70 (77.8%) of cases, and superficial in 20 (22.2%) cases. Twenty-six (28.9%) patients were managed with surgical resection alone (21 (30.0%) deep, 6 (30.0%) superficial). Two (2.2%) had chemotherapy during their treatment in addition to resection (2 (2.9%) deep, 0 (0%) superficial). Thirty-eight (42.4%) had radiation during their treatment in addition to resection (26 (37.1%) deep, 9 (45.0%) superficial). Twenty-four (26.7%) patients received both chemotherapy and radiation in addition to surgical resection (21 (30.0%) deep, 5 (25.0%) superficial) (Table 1). Twenty-five (27.8%) patients received flap reconstruction (i.e. local, regional, or free flap); of these 18 (25.7%) were patients with deep lesions and 7 (35.0%) were patients with superficial lesions (*p* = 0.41). Following the index surgical resection, a total of 33 (36.7%) patients had positive margins. Of deep lesions, 22 (31.4%) had positive margins compared to 11 (55.0%) superficial lesions (*p* = 0.068).

**Table 1:** Baseline demographics of the study population

Demographics	Frequency (%)
Age, in years 0-40 41-60 > 60	22 (24.4%) 32 (35.6%) 36 (40.0%)
Gender Male Female	49 (54.4%) 41 (45.6%)
Race White Black/African American Other/Unknown	77 (85.6%) 5 (5.6%) 8 (8.9%)
Histology Leiomyosarcoma Liposarcoma Myxofibrosarcoma Malignant fibrous histiocytoma Alveolar-type soft tissue sarcoma Angiosarcoma Synovial sarcoma Malignant peripheral nerve sheath tumor Other	8 (8.9%) 20 (22.2%) 16 (17.8%) 11 (12.2%) 1 (1.1%) 1 (1.1%) 9 (10.0%) 3 (3.3%) 21 (23.3%)
Location Upper extremity Lower extremity Hip/Pelvis	17 (18.9%) 63 (70.0%) 10 (11.1%)
Tumor depth Superficial Deep	20 (22.2%) 70 (77.8%)
Grade Low Intermediate High	27 (30.0%) 6 (6.7%) 57 (63.3%)
Tumor size, in cm 0-5 > 5	38 (42.2%) 52 (57.8%)
Margin status Positive Negative	33 (36.7%) 57 (63.3%)
Metastatic disease at presentation	5 (5.6%)
Managed with surgical resection only	26 (28.9%)
Received chemotherapy during treatment	2 (2.2%)
Received radiation therapy during treatment	38 (42.2%)
Received both chemotherapy and radiation	24 (26.7%)

**Fig. 1:**
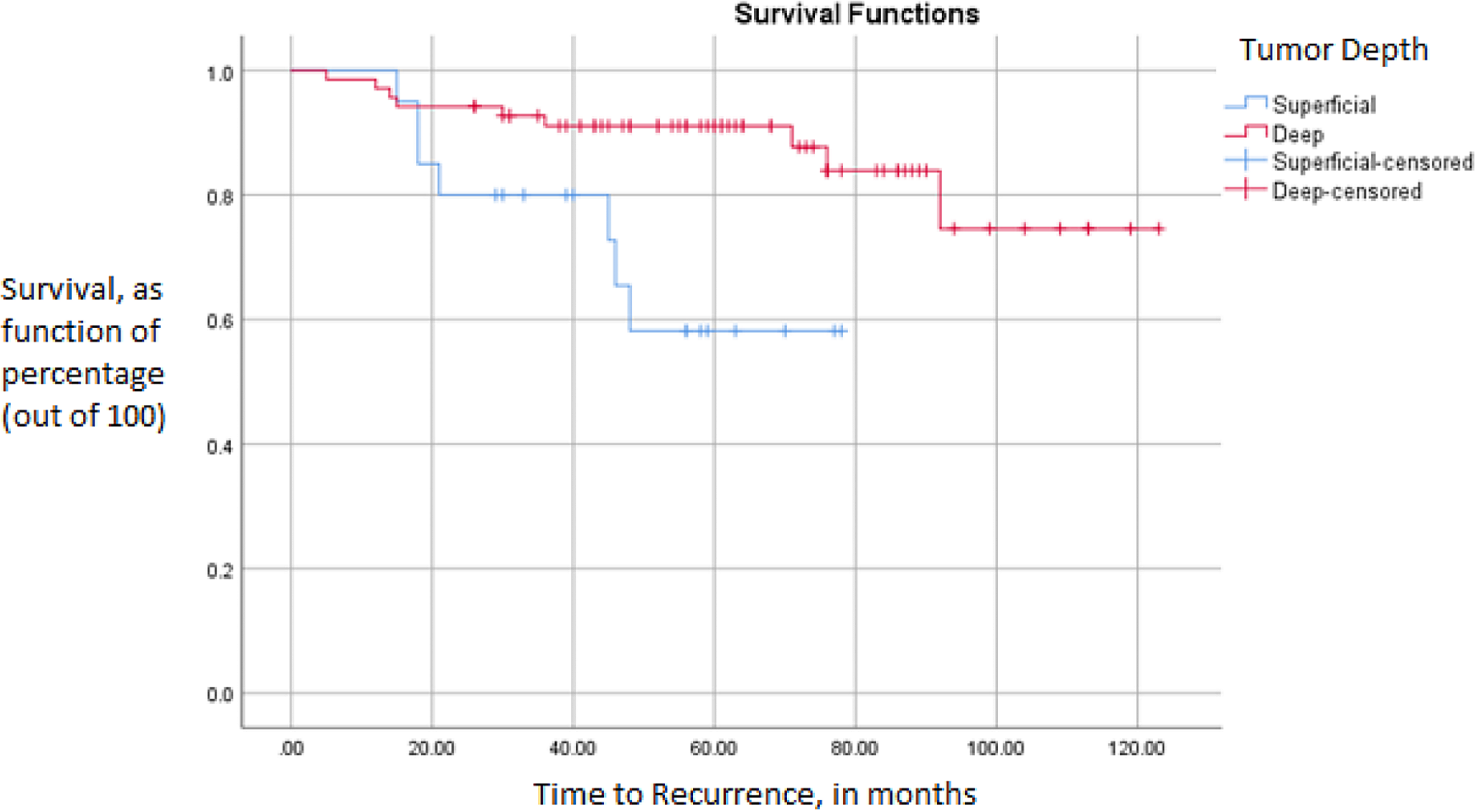
Kaplan-Meier survival showing recurrence-free survival (RFS), based on tumor depth. Rates of 5-year RFS: Deep (91.1%) vs. Superficial (58.2%); *p* = 0.006. Following a cox-regression hazard model, that utilized a backward step-wise approach (exclusion at *p* = 0.1), patients with higher tumor depth had lower odds of experiencing a local recurrence (HR 0.26 [95% CI 0.09–0.72]

A total of 17 (18.9%) patients had a local recurrence. Of patients with deep lesions, 10 (14.3%) had local recurrence compared to 7 (35.0%) with superficial lesions. Pearson-Chi square tests showed that postive margin status (*p* = 0.035) and superficial tumor depth (*p* = 0.037) were associated with local recurrence (Table 2). Unadjusted Kaplan-Meier survival analyses showed that deep tumors had a greater 5-year RFS (91.1%) vs. superficial lesions (58.2%) [log-rank p-value = 0.006) (Fig. 1). Following a cox-regression hazard model, that utilized a backward step-wise approach (exclusion at *p* = 0.1), patients with higher tumor depth had lower odds of experiencing a local recurrence (HR 0.26 [95% CI 0.09–0.72]).

**Table 2:** Patient characteristics and tumor factors associated with local recurrence

Demographics	No Local Recurrence	Local Recurrence	P-value
Age, in years 0-40 41-60 > 60	20 (27.4%) 26 (35.6%) 27 (37.0%)	2 (11.8%) 6 (35.3%) 9 (52.9%)	0.324
Gender Male Female	39 (53.4%) 34 (46.6%)	10 (58.8%) 7 (41.2%)	0.687
Race White Black/African American Other/Unknown	63 (86.3%) 3 (4.1%) 7 (9.6%)	14 (82.4%) 2 (11.8%) 1 (5.9%)	0.429
Histology Leiomyosarcoma Liposarcoma Myxofibrosarcoma Malignant fibrous histiocytoma Alveolar-type soft tissue sarcoma Angiosarcoma Synovial sarcoma Malignant peripheral nerve sheath tumor Other	7 (9.6%) 17 (23.3%) 12 (16.4%) 8 (11.0%) 0 (0%) 0 (0%) 8 (11.0%) 2 (2.7%) 19 (26.0%)	1 (5.9%) 3 (17.6%) 4 (23.5%) 3 (17.6%) 1 (5.9%) 1 (5.9%) 1 (5.9%) 1 (5.9%) 2 (11.8%)	0.158
Location Upper extremity Lower extremity Hip/Pelvis	13 (17.8%) 52 (69.9%) 9 (12.3%)	4 (23.5%) 12 (70.6%) 1 (5.9%)	0.685
Tumor depth Superficial Deep	13 (17.8%) 60 (82.2%)	7 (41.2%) 10 (58.8%)	0.037
Grade Low Intermediate High	22 (30.1%) 4 (5.5%) 47 (64.4%)	5 (29.4%) 2 (11.8%) 10 (58.8%)	0.642
Tumor size, in cm 0-5 > 5	31 (42.5%) 42 (57.5%)	7 (41.2%) 10 (58.8%)	0.923
Margin status Positive Negative	23 (31.5%) 50 (68.5%)	10 (58.8%) 7 (41.2%)	0.035
Metastatic disease at presentation	4 (5.5%)	1 (5.9%)	0.948
Managed with surgical resection only	22 (30.1%)	4 (23.5%)	0.769
Received chemotherapy during treatment	2 (2.7%)	0 (0%)	1.000
Received radiation therapy during treatment	29 (39.7%)	9 (52.9%)	0.415
Received both chemotherapy and radiation	20 (27.4%)	4 (23.5%)	1.000

## Discussion

We found that superficial soft tissue sarcomas of the extremity had higher rates of local recurrence than soft tissue sarcomas deep to the muscular fascia. The local recurrence rate of 35% for superficial lesions in our study is higher than the previously rates of 11–19.7% [2, 5, 18]. Superficial lesions also had a trend towards increased likelihood of having positive margins, which is a known risk factor for local recurrence [16, 28]. To our knowledge, an increased likelihood of local recurrence or having positive margins for superficial soft tissue sarcomas compared to deep lesions of the extremity has not been well demonstrated in the literature. A study by Biau et al. [4] showed initially increased risk of local recurrence for *deep* soft tissue sarcomas compared to superficial tumors, but there was little difference in cumulative probability of local recurrence in the long term. A series by Kainhofer et al. [16] did report significantly higher local recurrence for superficial tumors, but we are not aware of any other existing studies demonstrating this relationship between local control and tumor depth. We postulate that these findings may in part be due to the fact that superficial lesions are not bound by muscular fascia as deep lesions are. A recent study by Lee et al. [19] reports on the importance of the relationship that superficial soft tissue sarcomas have with the underlying deep peripheral fascia as seen on MRI scans. Invasion of the fascia was associated with worse disease free survival but not local recurrence in that study [19].

In our study, cases with positive margins had a 33.3% rate of local recurrence, which is comparable to that found in the literature [15, 16]. Some authors have suggested that positive or inadequate margins in superficial soft tissue sarcomas should receive further resection rather than adjuvant radiation to improve local control [2]. Although all our cases with superficial tumors and positive margins did ultimately receive a secondary surgery, we caution against defaulting to systematic immediate re-resection, as two-thirds of patients with positive margins will ultimately *not* experience local recurrence. In addition, recent data have also support the notion that a “watch and wait” strategy could be safe for certain patients with an *unplanned excision* of soft tissue sarcoma rather systematic re-excision [8]. Unplanned excision of soft tissue sarcoma inherently differs than positive margins following a planned resection by an oncologic surgeon, as the former may lead to higher residual disease and local recurrence [8]. Nevertheless, Decanter et al’s retrospective study [8] suggests that delaying re-excision to time of local relapse for an unplanned yet macroscopically complete excision of soft tissue sarcoma did not adversely affect metastatic relapse-free survival, overall survival, or rate of amputation.

Furthermore, the guidelines set forth by the National Comprehensive Cancer Network (NCCN) [34] as well as guidelines updated by specialists in the United Kingdom [7] contend both neoadjuvant and adjuvant radiation have a well established role. Radiotherapy may be avoided in cases with low-grade tumors and adequate resection as well as high-grade tumors if they are small, superficial, and resected with wide margins [7]. The NCCN [34] further provides specific recommendations for the role of radiotherapy based on American Joint Committee on Cancer (AJCC) stage [1]. Specifically, postoperative radiation was recommended for stage IA and IB lesions with surgical margins ≤ 1.0 cm and without an intact fascial plane. Both pre- and post-operative radiotherapy could be considered for stage II lesions, although resection alone may be adequate for small lesions if wider surgical margins are able to be achieved. Postoperative radiation was recommended for stage IIIA and IIIB disease, and chemotherapy could also be considered [34].

As superficial soft tissue sarcomas were more likely to have higher residual tumor levels by R-classification, it is possible that deep soft tissue sarcomas were managed more aggressively with regards to width of margins on resection. While many existing studies categorize margins as either positive or negative, some do report on specific width and quality of resection [10, 21]. Kainhofer et al. [16] found improved local recurrence rates with a minimal resection margin of 1 mm. Novais et al. [21] also found an inverse relationship between rates of local recurrence and resection margin, as margins of less than or equal to 2 mm had a 11.6% recurrence at 5 and 10 years follow up; margins between 2 mm and 2 cm had a 2.4% rate; and margins greater than 2 cm had a 0% recurrence rate. Similarly Dickinson et al. [10] also found increased width of resection resulted in lower local recurrence. However, increasing margins of resection could potentially lead to increased morbidity in a limb salvage surgery, and recommendations of width of resection is beyond the scope of this study. Nevertheless, it may be worth considering that superficial soft tissue sarcomas may have an elevated risk of local recurrence and certain cases should be managed in similar fashion to deep lesions.

A substantial limitation to this study is that this was a series of soft tissue sarcomas with heterogeneous histological types and anatomic locations. Soft tissue sarcomas are inherently rare malignancies, and it is challenging to investigate a large series of only a specific subtype of this disease. Patients presented to our institution and were diagnosed at a variety of different stages of disease. Although all patients in this series were management with surgical resection, the adjunctive treatments they received also varied widely as chemotherapy and radiation regimens were tailored specifically to the patient.

There are additional limitations to consider. This was a retrospective study at a single quaternary care center. Although we examined anatomic location and depth of invasion, we did not account for involvement of neurovascular structures. In addition, we did not control for metastases or lymph node involvement, and therefore conclusions drawn about survival and depth of invasion is limited. Selection bias may be present as we set a minimum of two-year follow up as our inclusion criteria. Therefore, the true overall and disease-free survival may not have been captured, as a subset of deceased patients would have not been included in our analysis. Specifically, patients presenting with aggressive malignancy potentially refractory to treatment may be underrepresented as they would more likely fall into this subset of patients who did not survive beyond two years after surgery. Small sample size also precluded adequately powered statistical analyses between certain subgroups. In this current study, cases with and without local recurrence had comparable overall survival. This data is mixed in the literature, but we speculate that recurrence for certain subtypes and grades of soft tissue sarcoma are likely more significant in terms of morbidity and mortality than others, and future studies should investigate this.

## Conclusion

These data suggest that local recurrence of soft tissue sarcomas of the extremity is associated with superficial depth of tumor. There was also a trend towards increased likelihood of positive resection margins for superficial tumors. Local recurrence was also associated with positive surgical margins at time of index resection. Given the elevated risk of local recurrence of these superficial soft tissue sarcomas managed by the standard of care, there may be value in approaching certain superficial lesions in a similar fashion as deep soft tissue sarcomas in order to curtail rates of recurrence and potentially secondary surgery in the future.

### Electronic supplementary material

Below is the link to the electronic supplementary material.

Supplementary Material 1
