# Supplementary material for: Local recurrence rates of superficial versus deep soft tissue sarcoma
Source: Arch Orthop Trauma Surg. 2024 Jun 8;144(7):2967–73. doi: 10.1007/s00402-024-05326-1 (PMC11319495; doi:10.1007/s00402-024-05326-1)
Supplement: Supplementary file 1 — Supplementary Material 1 [file 402_2024_5326_MOESM1_ESM.pdf]

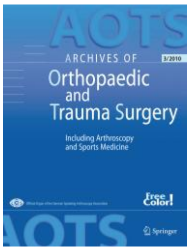

Springer
the language of science

Conflict of Interest and Disclosure Form

It is the policy of the Journal *Archives of Orthopaedic and Trauma Surgery* to ensure balance, independence, objectivity, and scientific rigor in the Journal. All authors are expected to disclose to the readers any real or apparent conflict(s) of interest that may have a direct bearing on the subject matter of the article. This pertains to relationships with pharmaceutical companies, biomedical device manufacturers or other corporation whose products or services may be related to the subject matter of the article or who have sponsored the study.

The intent of the policy is not to prevent authors with a potential conflict of interest from publication. It is merely intended that any potential conflict should be identified openly so that the readers may form their own judgements about the article with the full disclosure of the facts. It is for the readers to determine whether the authors' outside interest may reflect a possible bias in either the exposition of the conclusions presented.

The senior and the corresponding author will complete and submit this form to the Springer Journal's Editorial Office, Carsten Brall (Mr) on behalf of all authors listed below.

Article Title Local Recurrence Rates Of Superficial Versus Deep Soft Tissue Sarcoma

Authors James S. Lin M.D., Lisa Coleman M.S., B.S., Ryan T. Voskuil M.D., Azeem Malik MD, Joel L. Mayerson M.D., Thomas J. Scharschmidt M.D.

Please note that a conflict of interest statement is published with each paper.

I certify that there is no actual or potential conflict of interest in relation to this article. If any conflict exists, please define hereafter:

Conflict (if none, "None" or describe financial interest/arrangement with one or more organizations that could be perceived as a real or apparent conflict of interest in the context of the subject of this article):

None

CONDITIONS OF SUBMISSION

ORIGINALITY: Each author warrants that his or her submission to the Work is original and that he or she has full power to enter into this agreement. Neither this work nor a similar work has been published or submitted, nor shall it be submitted for publication elsewhere while under consideration by the *Archives of Orthopaedic and Trauma Surgery*.

AUTHORSHIP RESPONSIBILITY: Each author certifies that he or she has participated sufficiently in the conception and design of this work, intellectual content, the analysis of data, if applicable, and the writing of the work to take responsibility for the integrity and accuracy of the data. Each has reviewed the final version of the work, believes it represents valid work, and approves it for

publication. Moreover, the authors shall produce the data upon which the work is based for examination should the editors or their assignees request it.

Name (senior author) Thomas Scharschmidt

Signature [signature] Date October 12, 2022

Name (corresponding author) Thomas Scharschmidt

Signature [signature] Date October 12, 2022

Please send the completed and signed form to Carsten Brall (Mr): aots@springer.com

Springer

http://www.springer.com/journal/402

Archives of Orthopaedic and Trauma Surgery
Including Arthroscopy and Sports Medicine
Editors-in-Chief: Blauth, M.; Kröber, M. - Co-Editors:
Heyse, Th.J.; Unglaub, F.; Zantop, T.
ISSN: 0936-8051 (print version)
ISSN: 1434-3916 (electronic version)
Journal no. 402
